# Supplementary material for: Rapid whole-brain electric field mapping in transcranial magnetic stimulation using deep learning
Source: PLoS One. 2021 Jul 30;16(7):e0254588. doi: 10.1371/journal.pone.0254588 (PMC8323956; doi:10.1371/journal.pone.0254588)
Supplement: S1 File — (PDF) [file pone.0254588.s001.pdf]

Toward real-time electric field mapping in transcranial magnetic stimulation using deep learning

Guoping Xu[a,b], Yogesh Rathi[b], Joan A. Camprodon[c], Hanqiang Cao[d], Lipeng Ning[b]*

[a] School of Computer Sciences and Engineering, Wuhan Institute of Technology, Wuhan, Hubei, China [b]Department of Psychiatry, Brigham and Women's Hospital, Harvard Medical School, Boston, MA, USA

[c]Department of Psychiatry, Massachusetts General Hospital, Harvard Medical School, Boston, MA, USA

[d]School of Electronic Information and Communications, Huazhong University of Science and technology, Wuhan, Hubei, China

1. Volume conductor model (VCM) to generate E-field

We used the pipeline based on SimNIBS [1] to simulate E-field. The basic schema was shown in Figure S1, which included data preprocessing, segmentation, building volume conductor model, generating E-field by using the finite-element method et.al.

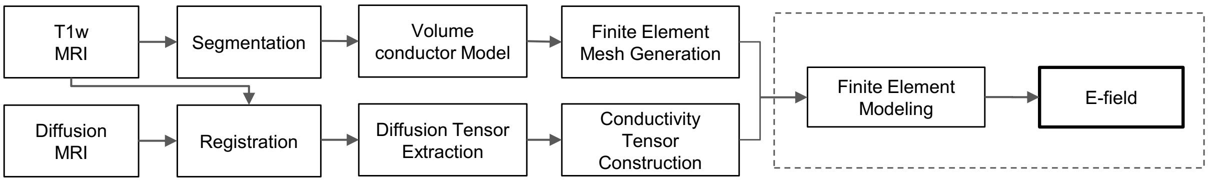

S1 Fig: The schema of VCM to generate E-field

Here, we use the *mri2mesh* command to reconstruct a tetrahedral head mesh from T1w structural MR images which will build a volume conductor model. Then, the command *dwi2cond* was employed to integrate conductivities from diffusion MR images into the VCM to calculate E-field by using the finite element method. It worth noting that we focus on the process that was shown in the dashed box in this study owing to time-consuming by FEM. In fact, it can be done offline to build VCM.

2. About training data for model *C-20* and *C-60*

It includes two parts of the training data for *C-20* and *C-60*: *primary eigenvector of the conductivity tensor* (PEC) and *dA/dt*. The PEC contains the anisotropic information from the conductivity tensor and the dA/dt caused by the selected coil means the peak temporal change of the vector potential. It worth noting that the PEC can be obtained offline and we need one PEC for each subject. One slice of the norm of PEC and dA/dt can be seen in Figure S2.

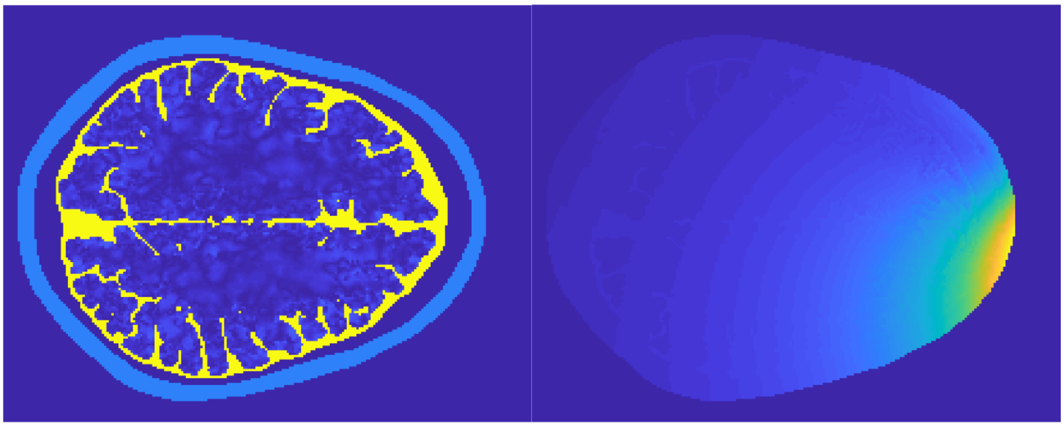

S2 Fig: One slice of the norm of PEC (Left) and dA/dt (Right)

It can be seen that the PEC has the anatomy structure from T1w MRI and the conductivity information extracted from diffusion MRI. Meanwhile, the dA/dt encoded the information on the location and direction of the coil. Moreover, it also has the property of the selected coil.

2. Further visualization of results

In this part, we will show some results that did not contain in main manuscript.

2.1 Results in volume space

We can see that the estimation of E-filed by VCM and *C-60* in Figure S1 respectively. It can be seen that the estimated E-field from VCM is a little sharper than from *C-60*, especially in the edge of the brain tissue.

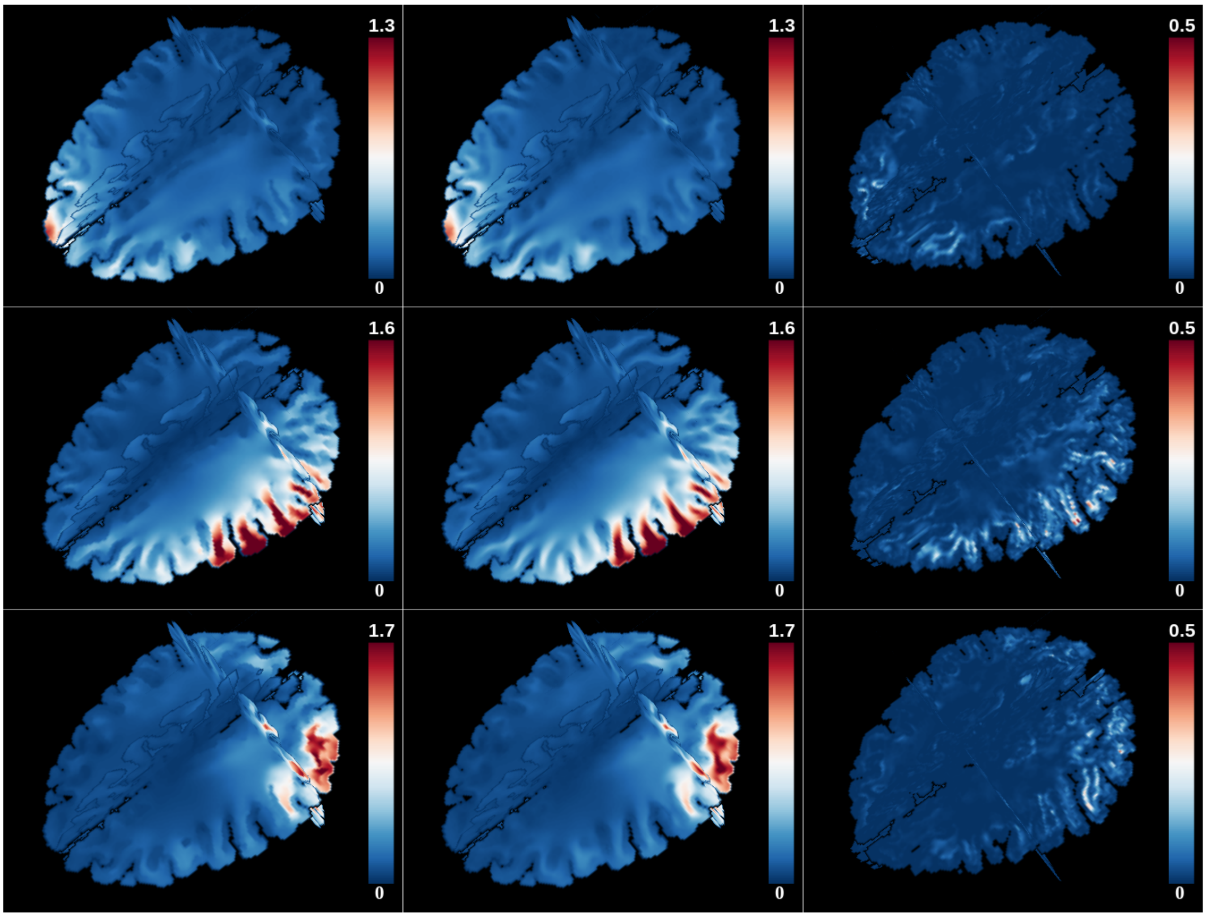

S3 Fig: The magnitude of E-field (mV/m) from VCM (the first column) and *C-60* (the second column), and absolute error (the last column) on the EEG positions *Fpz*, *C5* and *P5* of one subject in the volume level.

In Figure S4, it can be seen the distribution of topographical based on various metrics. It was shown that the performance tested by model *C-60* was much better than *T1-20* on the five metrics, e.g: TOC and EPD. Except for EPD, the other metric result demonstrated that there is some trend in the EEG positions by *T1-20* and *C-20*.

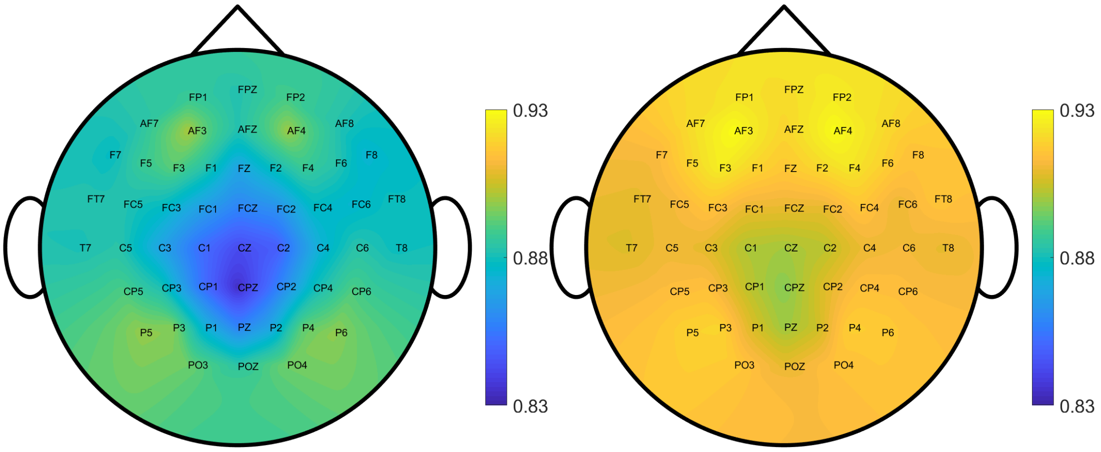

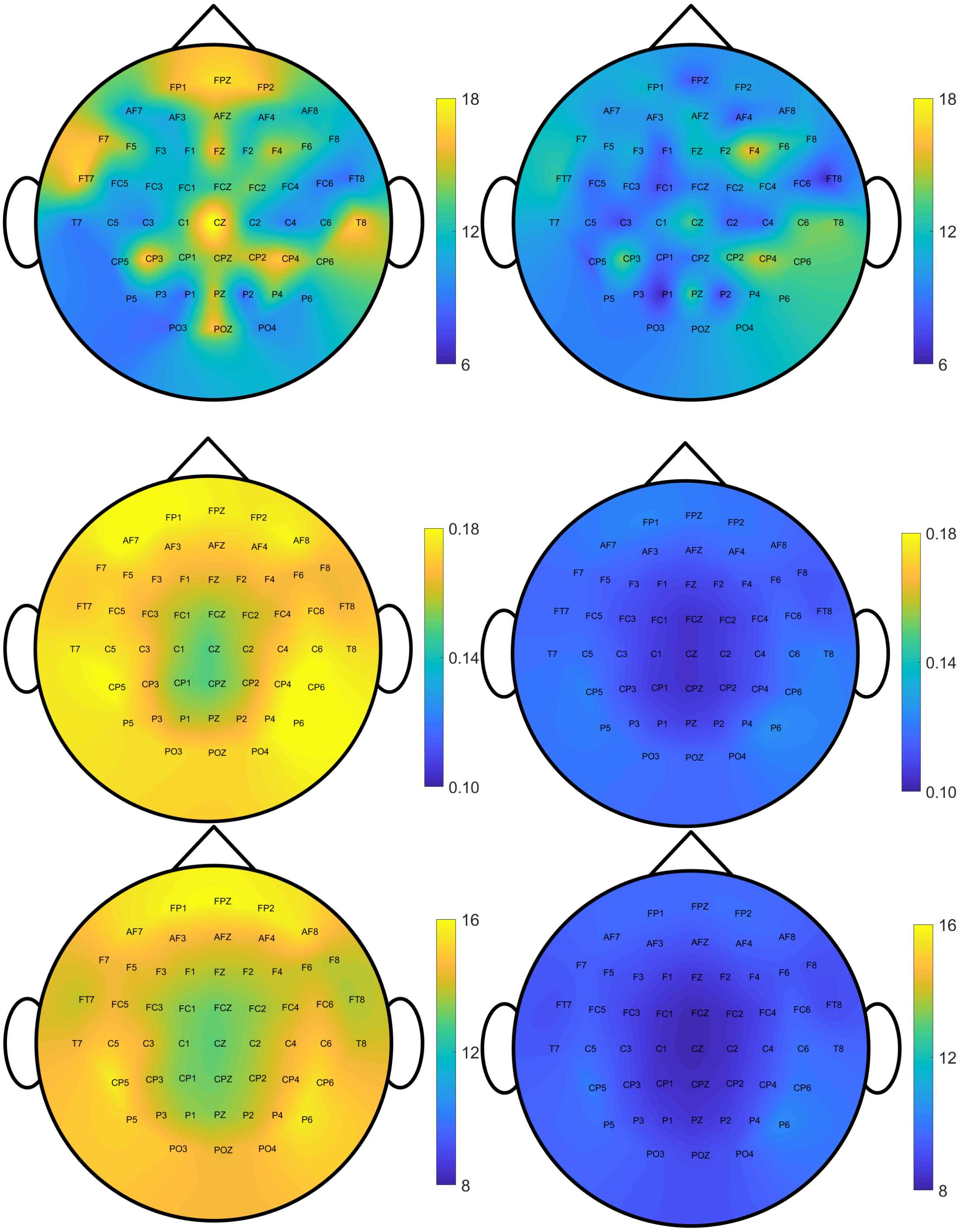

S4 Fig: The distribution of TOC, EPD, Correlation, MAE, MRE and MDE on EEG position with *T1-20* and C-60 models on volume space

In the Figure S5, it demonstrated the histogram by five metrics on 52 EEG positions.

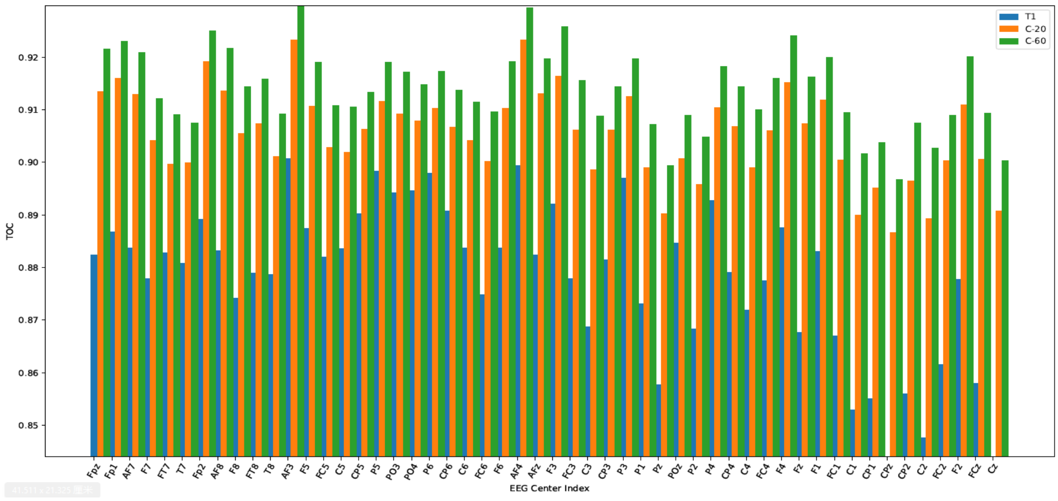

(a) The distribution of TOC on three models

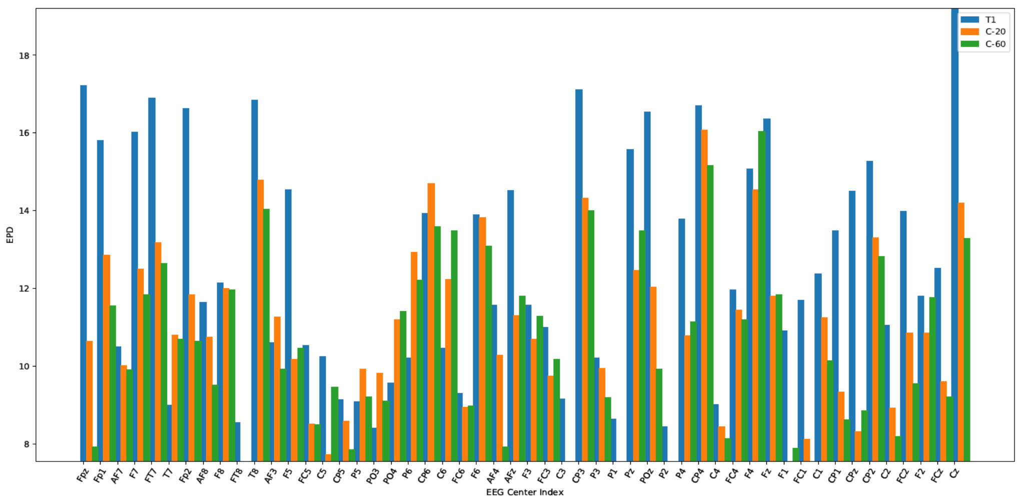

(b) The distribution of EPD on three models

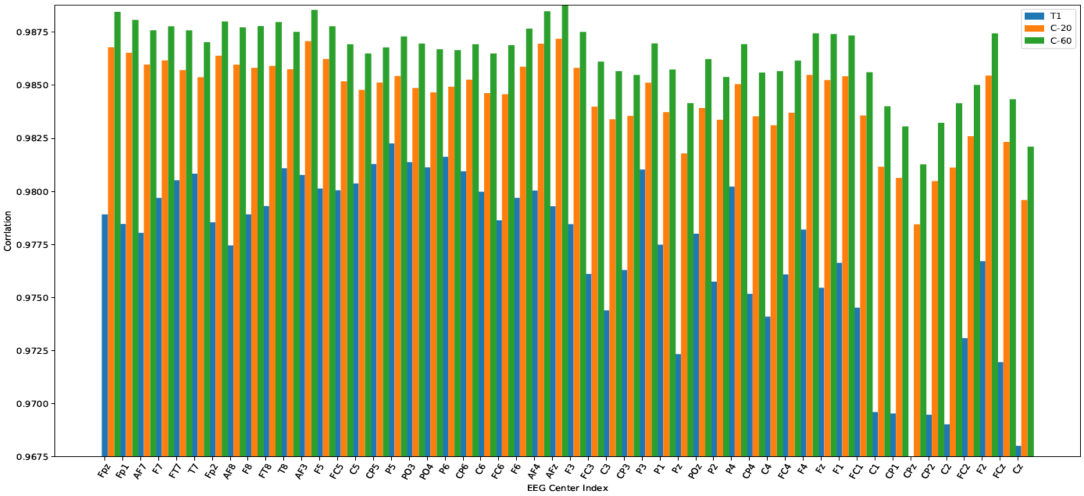

(c) The distribution of Correlation on three models

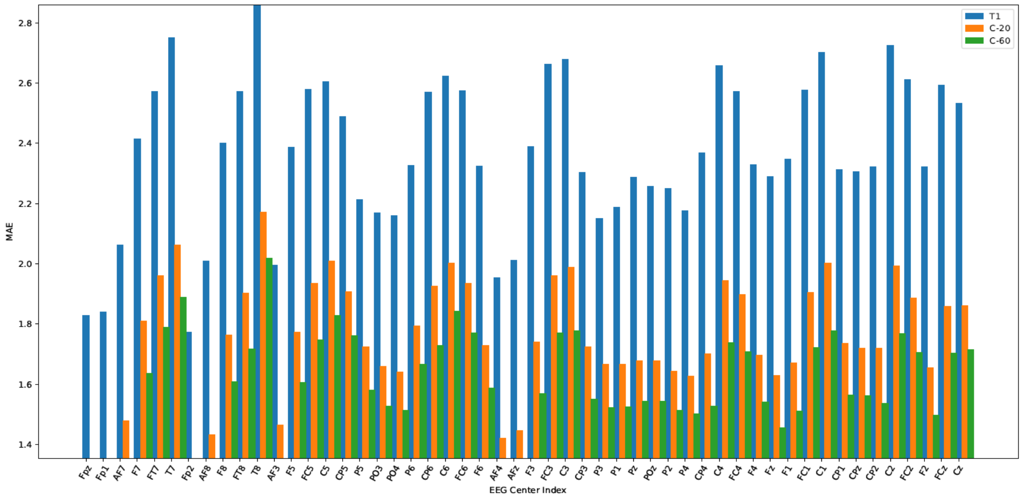

(d) The distribution of MAE on three models

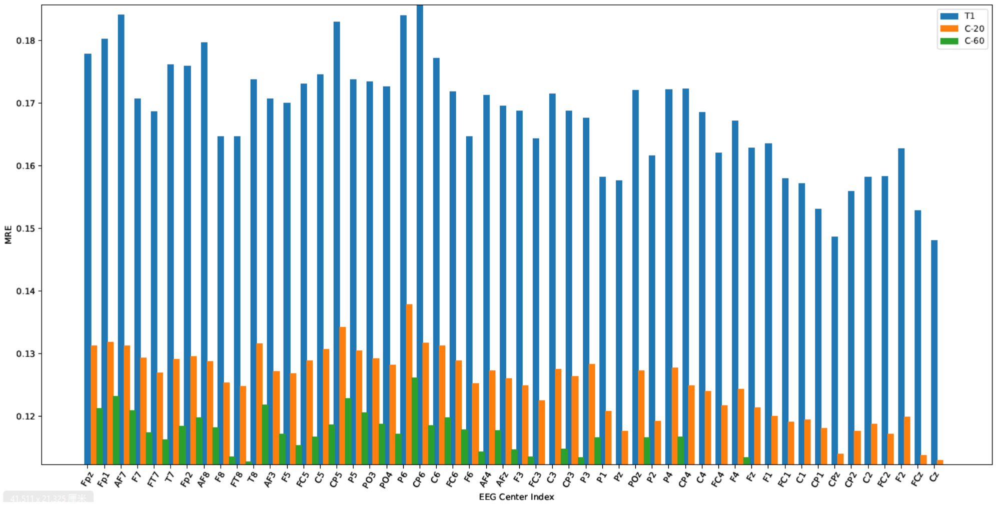

(e) The distribution of MRE on three models

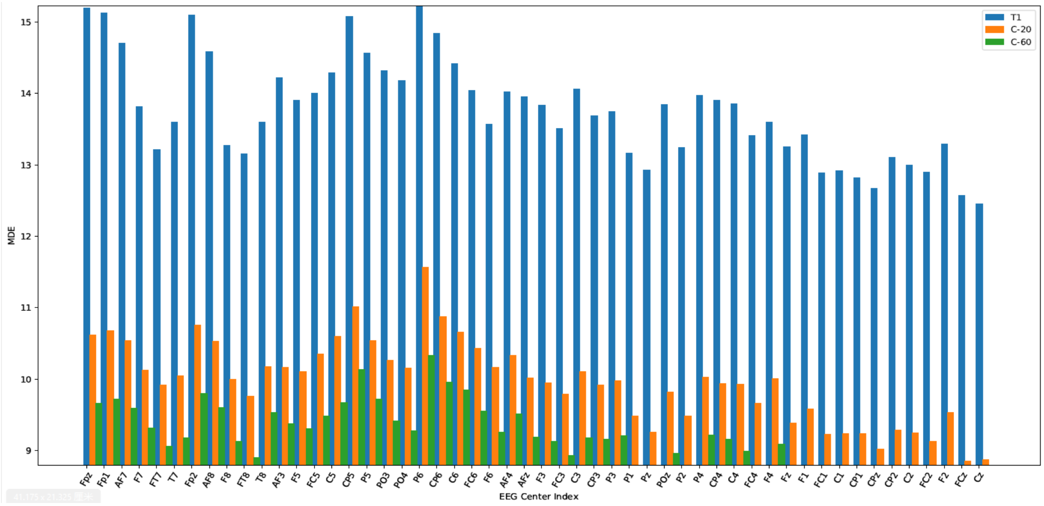

(f) The distribution of MDE on three models

S5 Fig: The distribution of various metric on three trained DNN models

2.2 Results in surface space

The figure S6 shows the estimation of E-filed in the rostral middle frontal surface from three different subjects with *C-60*. We can also find that it still has the high similarity between VCM and *C-60* except for the EEG positions, which indicates that the model can predict the E-field on any brain positions, although, all of the training data is sampled from EEG position.

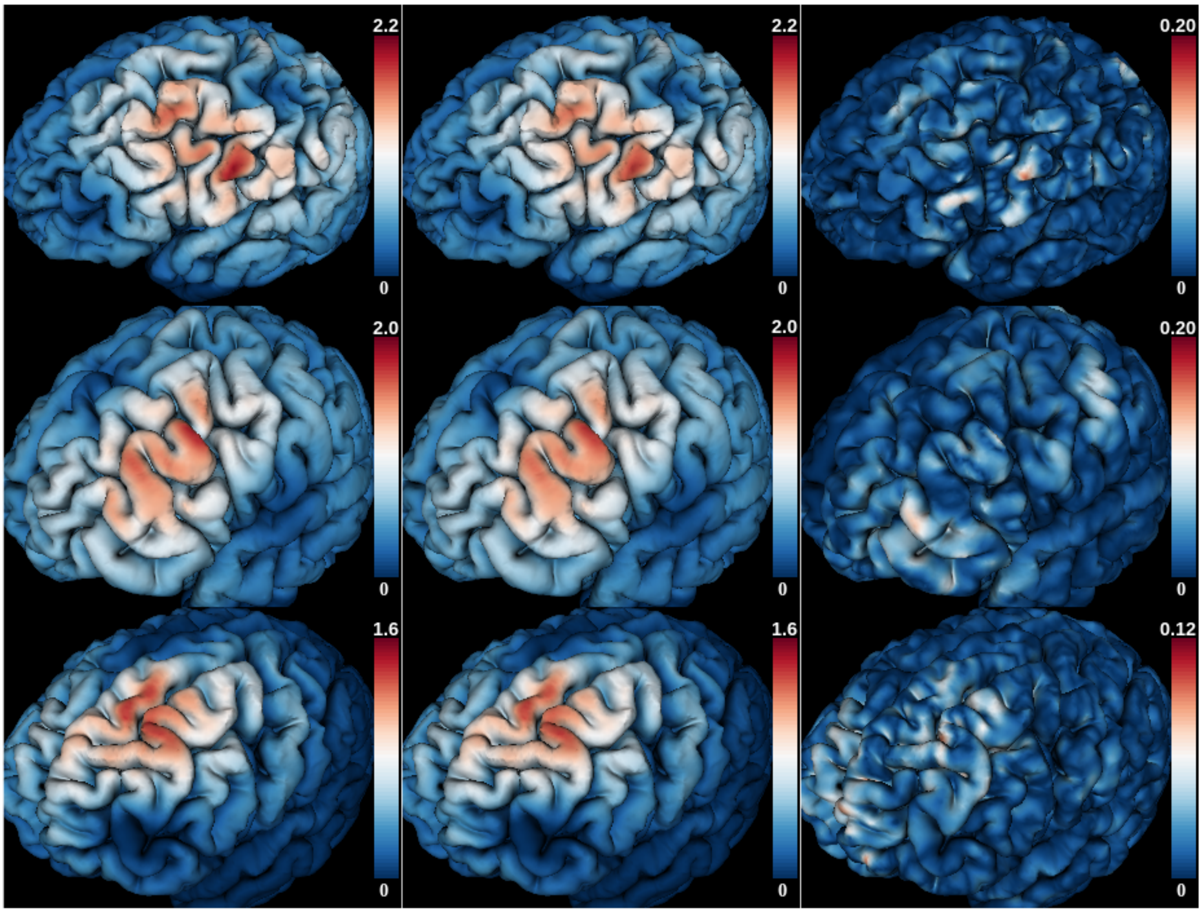

S6 Fig: The magnitude of E-field from VCM (the first column) and *C-60* (the middle column), and the absolute difference between VCM and *C-60* (the last column) on the rostral middle area of three subjects.

It can be seen that the E-field prediction from the trained network *C-60* is close to the result by using VCM, especially for the position of peak norm E. Although, all of the training data is sampled from EEG position, there is no much influence of the prediction performance on the region of rostral middle frontal.

Figure S7 shows the distribution of average dice coefficient, average correlation coefficients, mean relative error, mean absolute error, mean distance error and maximum peak value between VCM and *C-60* in the rostral middle area. We can see that most positions have high performance in the rostral middle area. Interestingly, some positions with relatively high errors usually happened in the edge of gray matter.

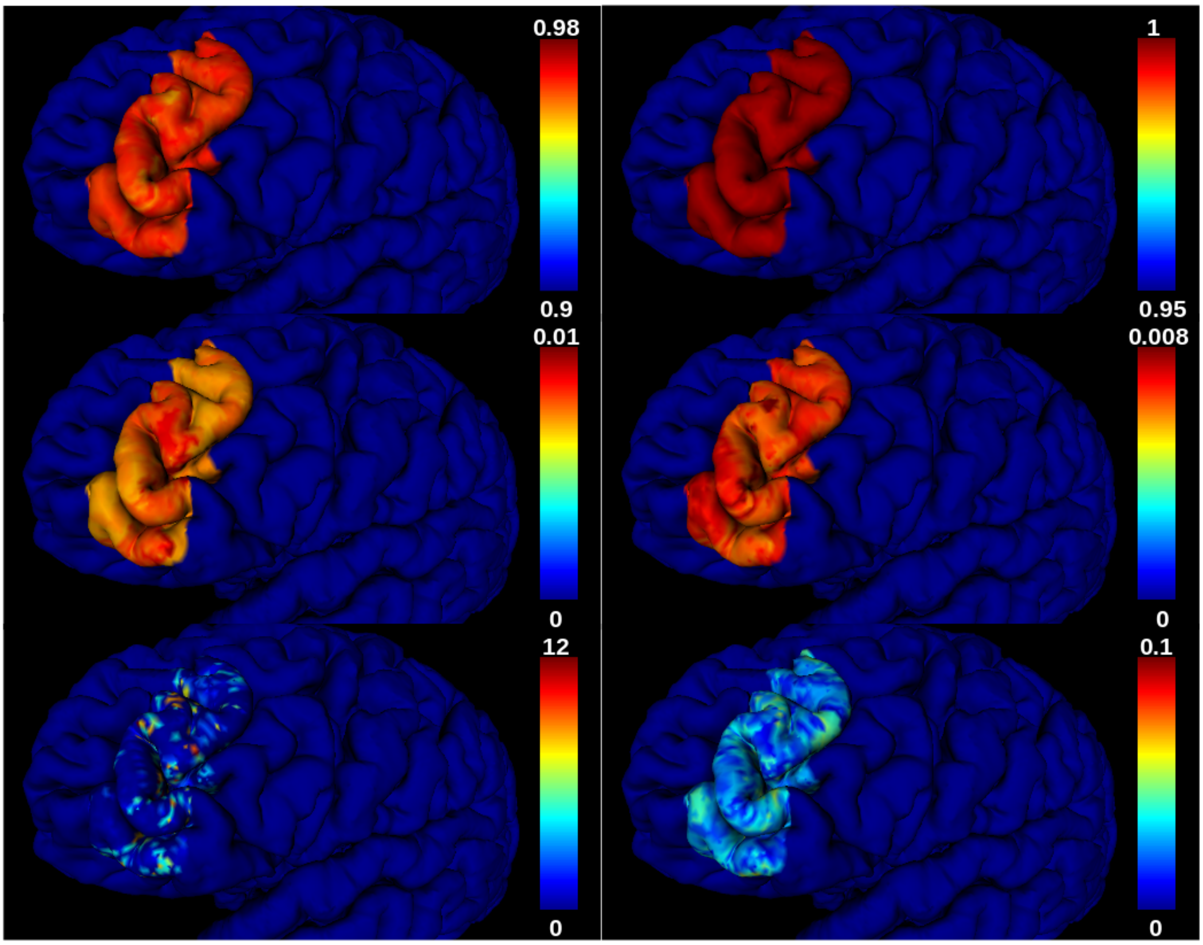

S7 Fig: The distribution of TOC, Correlation, MRE, MAE (mV/m), EPD (mm) and maximum peak value (mV/m) between VCM and *C-60* in the rostral middle area of a subject.

2.3 Performance tested on different epoch by three models

The figure S8 were shown the TOC, MAE, MRE and correlation on various epoch on five testing subject, in which each subject has 4056 samples. It can be seen that the performance will be improved as the epoch increased. Moreover, it is obvious that the model *C-20* is better that *T1-20*, which indicated the effectiveness to consider the conductivity in training our 3D-MS-Res-U-net.

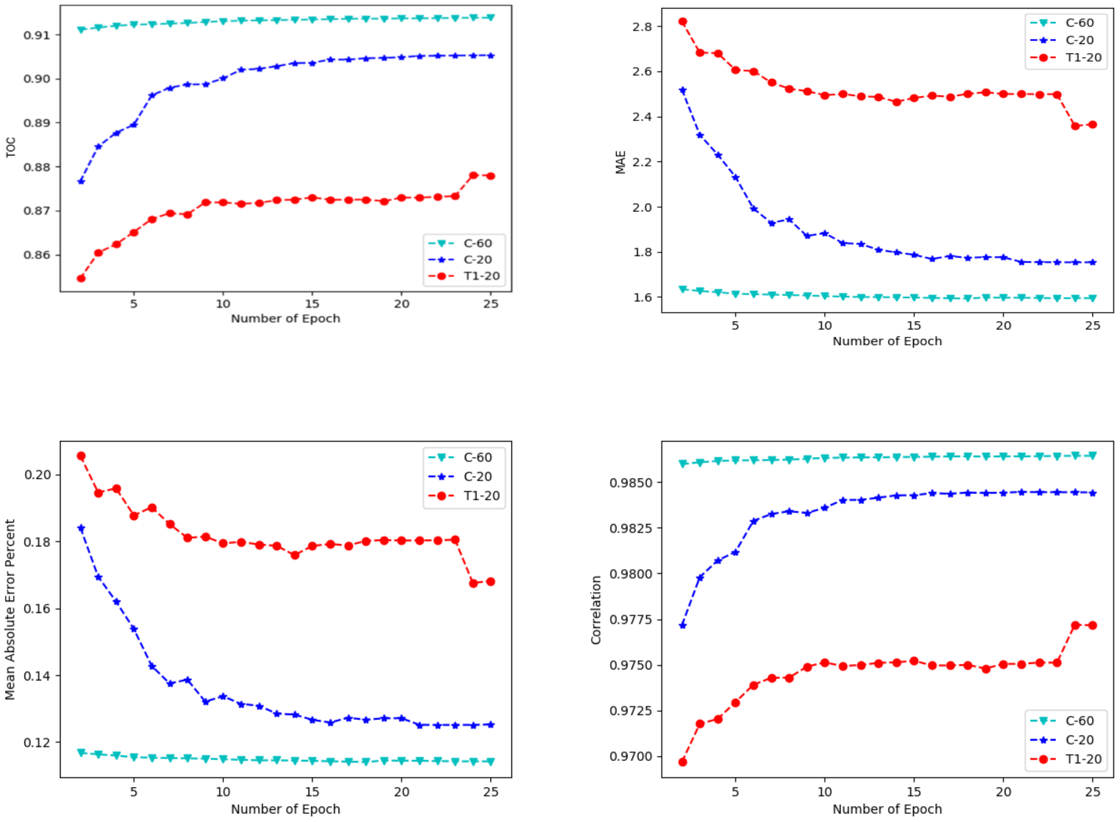

S8 Fig: The mean absolute error and correlation on testing data.

In this study, we introduced a volume-to-volume neural network, named 3D-MS-Res-U-net. The inputs are PEC and dA/dt with the size of 180*220*120*3 and 180*220*120*3, respectively. The output is the E-field, whose size is 180*220*120*3. The architecture of the network consists of 31 3D-convolution layers with batch normalization (BN)[2], leaky rectified linear units (LeakyReLU [3]), and up-samping. The details of the proposed neural network can be found in Table S8.

S8 Table: The detail of 3D-MS-Res-U-net architecture

Number	Layers	Feature Size	Parameters
0	Input	180*220*120*6	
1	Conv3d	180*220*120*16	2,592
2	LeakyReLU	180*220*120*16	
3	Conv3d	180*220*120*16	6,912
4	Dropout&LeakyReLU	180*220*120*16	
5	Conv3d	180*220*120*16	6,912

6	LeakyReLU&BN&LeakyReLU	180*220*120*16	
7	Conv3d	90*110*60*32	13,824
8	BN*LeakyReLU	90*110*60*32	
9	Conv3d	90*110*60*32	27,684
10	Dropout&BN&LeakyReLU	90*110*60*32	
11	Conv3d	90*110*60*32	27,684
12	BN*LeakyReLU	90*110*60*32	
13	Conv3d	45*55*30*64	55,296
14	BN*LeakyReLU	45*55*30*64	
15	Conv3d	45*55*30*64	110592
16	Dropout&BN&LeakyReLU	45*55*30*64	
17	Conv3d	45*55*30*64	110592
18	Dropout&BN&LeakyReLU	45*55*30*64	
19	Conv3d	23*28*15*128	221,184
20	BN&LeakyReLU	23*28*15*128	
21	Conv3d	23*28*15*128	442,368
22	Dropout&BN&LeakyReLU	23*28*15*128	
23	Conv3d	23*28*15*128	442,368
24	BN&LeakyReLU	23*28*15*128	
25	Conv3d	12*14*8*256	884,736
26	BN&LeakyReLU	12*14*8*256	
27	Conv3d	12*14*8*256	1,769,472
28	Dropout&BN&LeakyReLU	12*14*8*256	
29	Conv3d	12*14*8*256	1,769,472
30	BN&LeakyReLU&Upsample	24*28*16*256	
31	Conv3d	24*28*16*128	884,736
32	BN&LeakyReLU	24*28*16*128	
33	Conv3d	23*28*15*128	16,384
34	BN&LeakyReLU	24*28*16*128	
35	Conv3d	23*28*15*256	1,749,472
36	BN&LeakyReLU	24*28*16*128	
37	Conv3d	23*28*15*128	32,768

38	BN&LeakyReLU&Upsample	46*56*30*128	
39	Conv3d	46*56*30*64	221,184
40	BN&LeakyReLU	46*56*30*64	
41	Conv3d	45*55*30*128	442,368
42	BN&LeakyReLU	45*55*30*128	
43	Conv3d	45*55*30*64	8,192
44	BN&LeakyReLU&Upsample	90*110*60*32	
45	Conv3d	90*110*60*32	55,296
46	BN&LeakyReLU	90*110*60*32	
47	Conv3d	90*110*60*64	110,592
48	BN&LeakyReLU	90*110*60*64	
49	Conv3d	90*110*60*32	2048
50	BN&LeakyReLU&Upsample	90*110*60*32	
51	Conv3d	180*220*120*16	13,824
52	BN&LeakyReLU	180*220*120*16	
53	Conv3d	180*220*120*32	27,648
54	BN&LeakyReLU	180*220*120*32	
55	Conv3d	180*220*120*3	96
56	Conv3d	45*55*30*3	384
57	Upsample	90*110*60*3	
58	Conv3d	90*110*60*3	192
59	Upsample	180*220*120*3	
60	Conv3d	180*220*120*3	9
61	Output	180*220*120*3	

In this study, a volume-to-volume neural network architecture named 3D-MS-Res-U-net is introduced to predict the E-field. In the Table S1, it included the name of each layer and the output size of feature maps. It worth noting that feature maps from the 6th, 12th, 16th, and 22th layer are concatenated to 51th, 45th, 39th and 31th layer, respectively, by long skip connections. Moreover, additional deeply supervised layers from 56th and 58th are added into the U-net architecture, which help pass the multi-scale features extracted from early stage to later stage, see the Figure 1 in the main manuscript.

2.4 Performance demonstration on the video

In the video, we can see the point on the scalp that represents the coil, and the corresponding the E-field can be visualized at the same time. It was shown that the effectiveness of our proposed method.

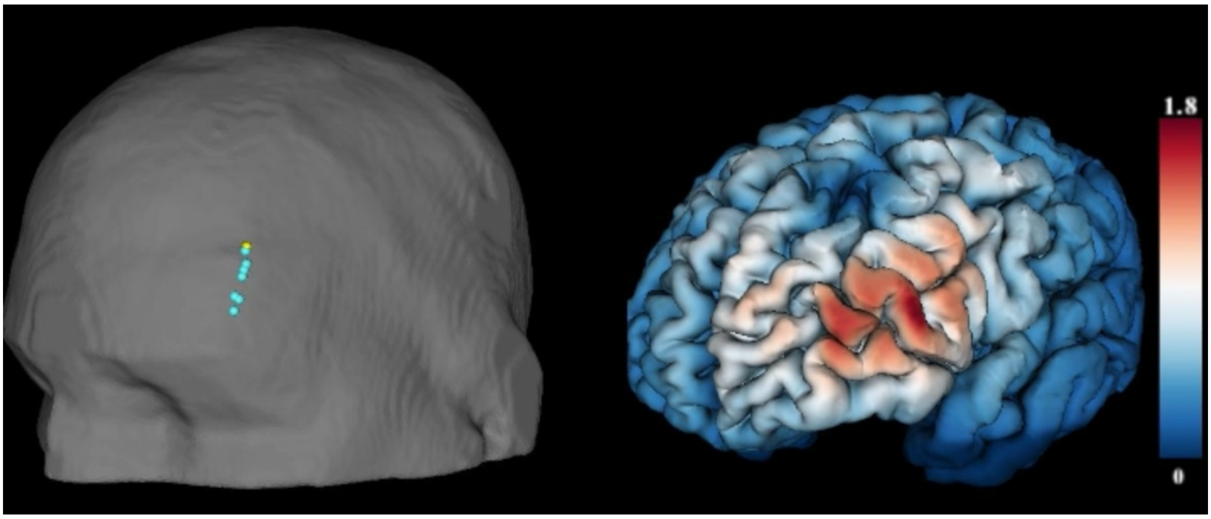

S9 Fig: One slice of the video. The maximum value of E-field is 1.8V/m in the video, and the yellow point means the current coil on the scalp.

Reference

[1] G. B. Saturnino, O. Puonti, J. D. Nielsen, D. Antonenko, K. H. Madsen, and A. Thielscher, "SimNIBS 2.1: a comprehensive pipeline for individualized electric field modelling for transcranial brain stimulation," in *Brain and Human Body Modeling*, Springer, 2019, pp. 3–25.

[2] S. Ioffe and C. Szegedy, "Batch normalization: Accelerating deep network training by reducing internal covariate shift," *arXiv preprint arXiv:1502.03167*, 2015.

[3] B. Xu, N. Wang, T. Chen, and M. Li, "Empirical evaluation of rectified activations in convolutional network," *arXiv preprint arXiv:1505.00853*, 2015.
