# Supplementary material for: Rapid whole-brain electric field mapping in transcranial magnetic stimulation using deep learning
Source: PLoS One. 2021 Jul 30;16(7):e0254588. doi: 10.1371/journal.pone.0254588 (PMC8323956; doi:10.1371/journal.pone.0254588)
Supplement: Attachment — Submitted filename: PlosOne_Response_v3.pdf [file pone.0254588.s003.pdf]

Dear Editor,

We thank you for the opportunity to submit a revision of our manuscript (#PONE-D-20-30151). We appreciate your generous approval to extend the deadline for the re-submission for us to complete the new experiments and revision. We are grateful for the reviewers' insightful and detailed comments which were helpful to improve the quality of this paper. We have individually addressed the reviewers' comments and concerns.

Our responses are given in blue and the revisions in the manuscript are highlighted in yellow. We hope that these changes meet and exceed your expectations for publication.

On behalf of myself and all the co-authors.

Sincerely,

Lipeng Ning, PhD.
Assistant Professor
Department of Psychiatry
Brigham and Women's Hospital
Harvard Medical School

Reviewer #1:

The manuscript left me with mixed feeling. It contains a good idea, but in its current state, it is unsuitable for publication.

First, the bad news: The authors use a DNN for approximate computation of a problem with known physics-based solution. They do not, however, compare their results to existing fast computation methods, but rather provide their 'accuracy' without any reference or comparison with either existing methods or requirements of the application the authors suggest the method to be used. The approach of using DNN to replace physics-based models is also questionable, see point 30 which paraphrases authors own critique of the earlier DNN work by (Yokota et al., 2019).

Thank you very much for your valuable suggestions. We have carefully revised the manuscript to address all your comments and concerns. We would like to clarify that the goal of the DNN-based method is not to replace FEM approaches. These FEM methods will always remain the "gold standard". However, once validated, the DNN-based methods can potentially be useful to accelerate the prediction of E-field where fast computation is required (e.g. fast targeting in a TMS clinic). Regarding the difference between our work and that of (Yokota et al., 2019), our method was developed to predict whole-brain electric field without restriction (or limitation) on the position or type of TMS coil. Further, the dimension of the predicted E-field of our method is 180x220x120x3 (i.e. whole brain) whereas the dimension of the output data by the method in (Yokota et al., 2019) is 72x144x24x1 (partial brain coverage). Thus, only a small subset of the brain region is predicted by the method of Yokota et al.

We have revised the manuscript to remove the comments on real-time prediction. We added the following sentence in Line 141 to address the difference from (Yokota et al., 2019):
Different from the method in [21], the proposed DNNs not only predict vector E-field maps but also predict it in the whole brain as opposed to scalar E-field maps in a small region. In particular, the dimension of the output data of the proposed DNNs is 180x220x120x3, whereas dimension of the output of the method in [21] is 72x144x24x1.

We added the following sentences in the Discussion in Line 472:
Moreover, the prediction speed is still slower than the fast quadrature method [18] and the DNN based method [17] though the predicted E-field by these methods is only in a smaller region of the brain. Further improvement in prediction speed in needed in situations when real-time visualization of E-field is required. For example, to predict 20 coil positions per second requires that the prediction time be reduced to less than 0.05 s. A potential method to improve the prediction speed is to reduce the field of view of the E-field or to only predict E-field on brain surfaces, which will be explored in our future work.

Then, the good news: their result with 'T1-20' model is interesting. This is an application where the DNN-based approach may shine. They did reach almost full accuracy of their approximate solver (with full input data) with limited input data. This should be the main result: with DNN, we can estimate the induced electric field of an anisotropic VCM without having to measure the anisotropy (which is expensive for subject-specific models). However, even with this change, the authors must perform additional comparisons. The accuracy of approximate DNN model must be compared to that of isotropic physics-based model. See my point 31. This alone is a reason I will currently have to suggest rejection, but most certainly with an option to resubmit.

We appreciate your helpful suggestion and the insightful viewpoint. Following your suggestion, we have trained a new DNN, named T1-iso20, that uses T_{1w} MRI and the dA/dt maps as input to predict the E-field simulated with isotropic models. The T-iso20 model was trained using the same amount of data as the T1-aniso20 and C-20 models. The first column of Table 1 in below summarizes the performances of the T1-iso20 model on brain surfaces. Overall, T1-iso20 has similar performance as the T1-aniso20 model which predicts anisotropic model-based E-field maps using T_{1w} MRI.

Table 1: Target overlapping coefficient (TOC), E-field peak distance (EPD) error [mm], Correlation, Mean Absolute Error (MAE) [V/m] and Mean Relative Error (MRE) and Mean directional error (MDE) between the VCM and DNNs on gray matter region of the whole brain for T1-iso20, *T1-aniso20,* C-20 *and* C-60 *and the rostral middle frontal area for* C-60 *on brain surfaces.*

Mean (SD)	EEG positions				Non-EEG positions
	T1-iso20	*T1-aniso20*	*C-20*	*C-60*	*C-60*
TOC	0.894(0.045)	0.906(0.024)	0.946(0.012)	0.956(0.009)	0.962(0.007)
EPD (mm)	3.9591(6.68)	3.736(6.055)	1.638(3.951)	1.394(3.663)	1.300(3.167)
Correlation	0.983(0.038)	0.986(0.008)	0.995(0.004)	0.997(0.004)	0.997(0.0015)
MAE	0.018(0.006)	0.017(0.005)	0.010(0.002)	0.008(0.001)	0.008(0.001)
MRE	0.154(0.059)	0.152(0.062)	0.089(0.014)	0.077(0.013)	0.077(0.009)

The results for volume space-based evaluations are provided in Table 2 below. T1-iso20 and T1-aniso20 models also have similar performances. We note that NRMSE in the table denotes the normalized root-mean-square error between vector E-field maps which is added following your comment #36. We evaluated the NRMSE for whole-brain E-field maps and the E-field around the target regions. Similarly, we computed the mean directional error (MDE) for both whole-brain E-field and the target regions.

Table 2: Performance of the models using independent HCP datasets. The evaluation metrics include the target overlapping coefficient (TOC), the E-field peak distance (EPD) error, the Correlation, the Normalized root-mean-square error (NRMSE) and the Mean directional error (MDE) between the VCM and DNNs on gray matter region of the whole brain for *T1-iso20*, *T1-aniso20*, *C-20* and *C-60* and the rostral middle frontal area for *C-60* in the volume space.

	EEG positions				Non-EEG positions
	T1-iso20	*T1-aniso20*	*C-20*	*C-60*	*C-60*
TOC	0.874(0.033)	0.878(0.027)	0.905(0.014)	0.914(0.012)	0.931(0.008)
EPD (mm)	8.225(8.539)	8.709(8.789)	5.588(7.112)	4.977(6.546)	3.842(5.671)
Correlation	0.978(0.026)	0.977(0.008)	0.984(0.003)	0.986(0.003)	0.989(0.001)
NRMSE	0.264(0.0744)	0.277(0.083)	0.205(0.019)	0.187(0.017)	0.217(0.019)
NRMSE (target)	0.154(0.033)	0.153(0.031)	0.107(0.010)	0.097(0.009)	0.104(0.007)
MDE	13.455(3.338)	13.770(4.039)	9.969(1.133)	9.117(0.977)	10.814(0.930)
MDE (target)	6.688(2.545)	6.605(1.299)	4.841(0.426)	4.474(0.368)	4.957(0.412)

Further, to ensure that the model is really robust to change of coil, the tested coil model should be fundamentally different from the teaching coil model. Now they are two figure-of-eight coils with fairly similar sizes (one step down in size). The testing coil could be, e.g., a circular coil or an H-coil.

Following your suggestion, we applied the C-60 model to predict the E-field simulated using the Magstim-70mm-circular coil in addition to the previously used MagVenture-MC-B70 coil. The results are summarized in Table 3 below.

Table 3: Performance of the C-60 model using different coils for the HCP and Ernie dataset. The evaluation metrics include the Target overlapping coefficient (TOC), the E-field peak distance (EPD) error, the Correlation, the Normalized root-mean-square error (NRMSE) and the Mean directional error (MDE) in the volume space.

		Magstim-70mm-Fig8	*MagVentur-MC-B70*	*Magstim-70mm-Circ*
TOC	HCP	0.914(0.012)	0.909(0.012)	0.890(0.012)

	Ernie	0.905(0.011)	0.895(0.015)	0.8858(0.014)
EPD (mm)	HCP	4.977 (6.546)	4.789(6.636)	6.257(6.174)
	Ernie	7.591(8.588)	7.236(8.951)	18.451(12.620)
Correlation	HCP	0.986(0.003)	0.986(0.003)	0.985(0.003)
	Ernie	0.982(0.004)	0.981(0.004)	0.9806(0.005)
NRMSE	HCP	0.187(0.017)	0.312(0.045)	0.244(0.056)
	Ernie	0.3375(0.117)	0.511(0.194)	0.1123(0.3503)
NRMSE (target)	HCP	0.097(0.009)	0.116(0.012)	0.105(0.016)
	Ernie	0.121(0.016)	0.156(0.018)	0.1418(0.019)
MDE	HCP	9.117(0.977)	11.534 (1.966)	12.432(3.357)
	Ernie	15.985(4.415)	18.382 (9.776)	18.3903(5.973)
MDE (target)	HCP	4.474(0.368)	4.965(0.494)	4.407(0.592)
	Ernie	5.474(0.889)	5.935(1.096)	5.2675(1.0954)

Figure 4 illustrates the simulated and the predicted E-field maps by the C-60 model for the three types of TMS coils placed at the same position of a testing subject of the HCP dataset. The E-field maps corresponding to the Magstim-70mm-Fig8 and MagVenture-MC-B70 coils have similar E-field distributions in target regions because the two coils have similar structure and size. But the results for MagVenture-MC-B70 have higher prediction error especially for brain areas outside of the target areas. The last row of Figure 4 shows that the predicted E-field map for the Magstim-70mm-Circular coil has similar distributions as the simulation results, though the C-60 was trained based on the Magstim-70mm-Fig8 coil.

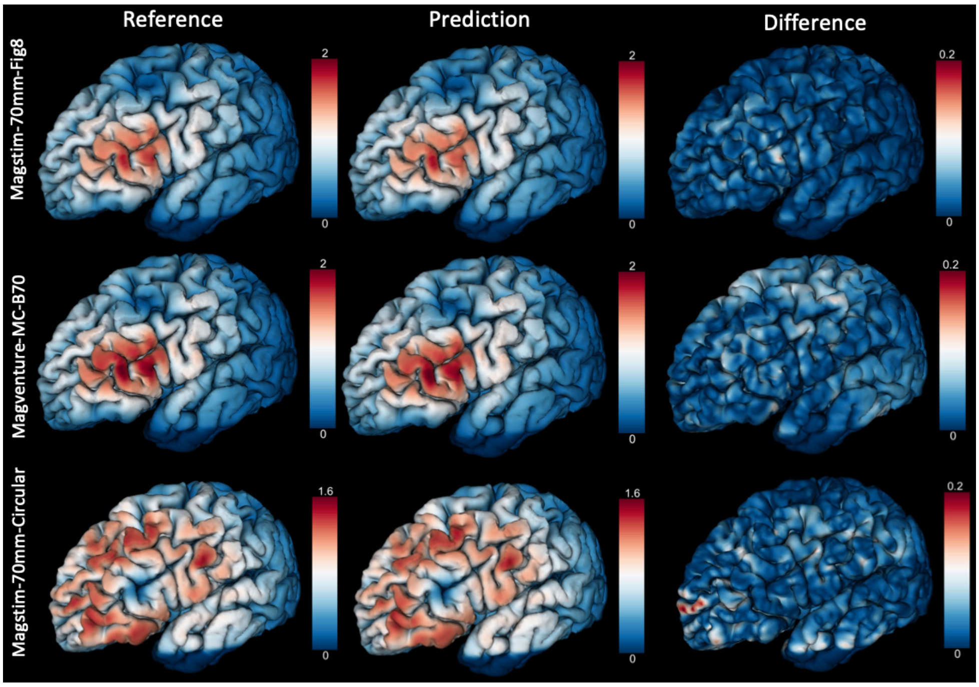

Figure 4: The magnitude [V/m] of E-field from VCM (the first column) and C-60 (the middle column), and the absolute difference between VCM and C-60 (the last column) for three types of TMS coils placed at the same position of a testing subject of the HCP dataset.

We hope our effort on the new experiments and the results are helpful to address your concerns and meet your expectation on the revision.

> *Finally, it seems that the authors have used the SimNIBS with incorrect parameters (omit the T2 images, which is needed to correctly predict the thickness of the skull in the modelling pipeline). Thus, much of the results need to be re-computed.*

Thanks for pointing out this limitation of our approach. In SimNIBS preprocessing, both T_{1w} and T_{2w} MRI can be used by FreeSurfer to improve brain parcellation results and provide more accuracy E-field maps. Thus, it is potentially useful to use both T_{1w} and T_{2w} in DNN models to improve the prediction of E-field maps. But to examine the difference between the results based on only T_{1w} and using both T_{1w} and T_{2w} MRI requires several months of computation time for simulating 101,400 E-files maps and several months for model training. Thus, we will explore it in our future work. We also have added the following sentences in Line 484 about the limitations:
Second, the trained DNN models not only depend on the type of coils, imaging protocols but also the data processing methods. In particular, tissue segmentations in this study were obtained based on T_{1w} MRI, but more accurate results can be obtained by using both T_{1w} and T_{2w} MRI. Thus, further development and training of the DNN models are needed to integrate different tissue segmentation approaches for more accurate prediction results.

> *I will begin with minor technical observations:*

*The authors, for no apparent reason, limit availability of their data. Their data availability statement has arbitrary requirement of "The data that support the findings of this study are available from the corresponding author upon *reasonable* request." The authors provide no legal or ethical reason to limit access in this manner.*

We shared the trained models and the python scripts used for training and evaluation via our Github link: https://github.com/LipengNing/Efield, which was added to the data availability statement. We note that simulated and predicted E-field maps take more than 40 TB storage space which makes it possible to be shared directly. Since all the training and testing MRI data are based on the public database from Human Connectome Project and the Ernie dataset provided by SimNIBS, the results can be reproduced without sharing the original data.

I further wonder why this work is submitted to PLOS ONE and not into some of the more specialized journals on neurostimulation methods.

We had submitted the manuscript to other specialized journals related to neurostimulation, yet the handling editor considered the topic was not appropriate for that journal. We believe PLOS ONE is a suitable journal for this paper since it has more diverse audiences.

The abstract:

1. *First sentence: TMS, in addition to being a 'neuromodulation' method is a 'neurostimulation' method. The latter is more descriptive, as neuromodulation is possible also with the subthreshold stimulation methods.*

Thanks. We changed "neuromodulation"' to "neurostimulation" in the first sentence following your suggestion.

2. *The second sentence is misleading: "Due to the complex structure of the brain and the electrical conductivity variation across different tissues, it is difficult to exactly identify the brain region stimulated by TMS, which is important to improve the treatment efficacy and understand the underlying mechanism."*
   Even based on this work, this computation is very straightforward with readily available toolkits to compute this. Further, this work, at best tries to reproduce the accuracy of these existing methods. Hence, calling this problem difficult is misleading.

Thanks. We have revised the sentence as following: Due to the complex structure of the brain and the electrical conductivity variation across subjects, identification of subject-specific brain regions for TMS is important to improve the treatment efficacy and understand the mechanism of treatment response.

3. *The "relative long computation time" is an incorrect claim. Realistic VCM models have been solved in under 25 ms, a further factor of 10 faster than the approximate*

solver shown in this work. And, compared to fast FEM implementations (such as the 3-second computation in Yokota et al., 2019) the authors speedup does not make a fundamental difference.

This is because the authors fail to define what they mean with "real-time computation". Their computation speed is much lower than in any of the previous three methods for 'real-time computations' (the local spherical models, the previous magnitude-only isotropic-VCM DNN, or a hardware-accelerated, fast-quadrature, isotropic-5C-VCM BEM).

The authors current computation speed of 240 ms = 4.2 computations per second = 4.2 frames per second is still too slow for neuronavigation (where the operator needs a smooth feedback, at minimum at least 15 frames per second, i.e. below 67 ms, and ideally >30 or >60 frames per second, i.e., 33 ms and 17 ms, respectively). The spherical model rans readily at these speeds, as did the earlier DNN which computed the fields in 10–30 ms (Yokota et al., 2019), or the real-time BEM which computed the fields in 15–23 ms based on it computing 46–65 coil positions per second (Stenroos & Koponen, 2019). In the current performance bracket, the model does not provide a quantitative improvement over the fast FEM solvers (about 3 s, not the number suggested in the work at 30-60 s), but is quantitatively too slow for the suggested 'real-time' application by a factor of at least 4.

Thanks for pointing out this problem. We have completely revised the manuscript to remove the "real-time" descriptions of this approach. In Discussion and Conclusions, we added the following paragraph about limitations on the prediction speed compared to prior works.

The prediction of a whole-brain E-field volume using the trained neural networks took about 0.24 s. In practice, additional time is needed to apply rigid transformation to the dA/dt map according to the coil position which is expected to take much shorter computation time. Moreover, the prediction speed is still slower than the fast quadrature method [18] and the DNN based method [17] though the predicted E-field by these methods have relative lower dimensions. Further improvement in prediction speed in needed in situations when real-time visualization of E-field is required. For example, to predict 20 coil positions per second required the prediction time should be reduced to less than 0.05 s. A potential method to improve the prediction speed is to reduce the field of view of the E-field or to only predict E-field on brain surfaces, which will be explored in our future work.

We would like to point out that our method was developed for whole-brain E-field prediction for different positions of coil position. The dimension of output data of our method is 180x220x120x3 whereas the dimension of the output data by the method in (Yokota et al., 2019) is 72x144x24x1, which is the main reason for difference in prediction speed.

4. *Abbreviation FEM (finite element method) should be defined before its first use in the abstract.*

It is introduced in Line 25.

Introduction:

5. *Line 42: the TMS coil current is pulsed, not oscillating (which suggests a continuous stimulation instead of discrete pulses). Even at highest repetition rates, the duty cycle of TMS is less than 1%, and typically it is much less than 0.1%.*

We changed oscillating to 'pulsed' in Line 40.

6. *Line 52: the "multi-sphere method" is commonly known as "local sphere model". This is also the case for the provided reference by the authors. Further, instead of the text-book chapter, the local spherical model should probably be attributed to its source, based on the book (Ilmoniemi et al., 1996), or as that work, similarly to the cited text-book chapter, is not readily available, their journal publication (Ilmoniemi et al., 1999).*

We changed "multi-sphere model" to "local sphere model" and added the journal publication (Ilmoniemi et al., 1999), i.e., reference [6], in Line 49.

7. *Line 53: TMS-specific BEM should be attributed to more correct, older references such as (Salinas et al., 2009) or (Nummenmaa et al., 2013).*

We added both references in Line 49, which are now references [8] and [9]. Thanks for the suggestions.

8. *Line 54: TMS-specific FEM, similarly to above, should be attributed correctly (Miranda et al., 2003).*

The suggested reference was added as [10] in Line 50.

9. *Line 54: remove word "sophisticated", as it does not apply only to FEM VCM. The BEM VCM are equally, or sometimes even more sophisticated given the recent advanced in fast-multipole methods.*

The word "sophisticated" was removed.

10. *Further, related to the claim "but also anisotropic tissue conductivity, which cannot be done by other methods" (line 56), the authors glossed over FDM methods which can model the anisotropy (e.g., De Geeter et al., 2011). The authors further failed to justify why the anisotropy in particular is of such a high importance (given the uncertainty conductivity values, for both isotropic and anisotropic case).*

The sentence in Line 50 was revised to below: the FEM method is based on a volume conductor model (VCM) of head tissue which not only is able to characterize complex tissue structure, brain geometry but also anisotropic tissue conductivity that can potentially improve the model precision especially in white matter regions [13][14].

11. *Line 66: BEM is not an accelerating algorithm!*

We revised the sentence in Line 61 as below: To overcome the limitations, several computation algorithms [20][21][22]have been developed to reduce the simulation time.

12. *Further, the sentence in line 66 should contain both (Stenroos & Koponen, 2019) and (Yokota et al., 2019), the existing real-time solvers.*

The suggested references were added as [22] and [21] in Line 61.

13. *Finally, I do not see any fundamental reason why (Laakso & Hirata, 2012) could not include also the anisotropy. It is an FEM after all. They likely followed the common convention in TMS-stimulation literature of omitting the anisotropy as we do not really know the anisotropic conductivity tensor. (As the authors also admit when they slightly latter use one of the three rules of thumb, none of which is even based on the physics, of deriving the anisotropic conductivity values from the DTI data and the not-that-accurately-known isotropic conductivity value.)*

We removed the part of sentence that the method (Laakso & Hirata, 2012), i.e. [20] does not include anisotropic conductivity.

14. *Line 74: The authors again claim their method capable of real-time simulation in clinical and research setting despite it being too slow for the suggested real-time neuronavigation use.*

We have revised the sentence and removed the word "real-time".

15. *Line 85: Overselling, every existing physics-based computation method (spherical models, FEMs, FDMs, BEMs) predicts the three-dimensional vector E-field: "In particular, our approach predicts the three-dimensional vector E-field instead of its magnitude." The sole method that does not, is the previous DNN method by (Yokata et al., 2019).*

We have revised this sentence. We had emphasized that our method is used to overcome the limitations of the previous DNN based method in (Yokata et al., 2019). Below are the revised sentences that are now in Line 76:

In this work, we propose a new deep-learning framework that overcomes the limitations of previous method in [21]. First, our approach predicts the three-dimensional vector E-field

instead of its magnitude. Second, our method can be used to predict E-field with the TMS coil placed at different positions over the whole brain. Third, our method uses the change of vector potential, i.e., the dA/dt map, of the TMS coil as input to predict E-field. Thus, the trained DNN can be applied to predict E-field for different types of coils.

16. *Line 88: Advertising a weakness of the method as its strength. The 'C-20' and 'C-60' require much more time-consuming and costly DTI dataset of each individual TMS subject, compared to just T1 (and in most cases T2 for best quality) MRI. It is not a strength to require the extraordinarily high-resolution input files of the HCP, but a limitation: "Furthermore, both diffusion-MRI based conductivity tensors and T1w MRI, which integrate anisotropic conductivity of brain tissues, are used to predict the E-field."*

We agree that the need for diffusion MRI is a limitation in applications. We have revised the sentences in Line 81 to below:
We have developed four deep neural networks (DNNs) that use different types of imaging data to predict vector E-field. Similar to the method in [21], the first two DNNs were trained based on T1w MRI images to predict E-field simulated using isotropic or anisotropic tissue conductivity tensors, respectively. The other two DNNs take the anisotropic tissue conductivity maps derived from diffusion MRI as the input to predict the E-field maps. By comparing the prediction results of the four DNNs, we can examine if the additional information provided by diffusion MRI can enhance the prediction accuracy.

17. *Line 90: Overselling similarly to point 15. All but the previous DNN method allow straightforward change of the coil model.*

The corresponding sentence "the magnetic field of the TMS coil was used as a part of input instead of using only the position and orientation" is now removed.

18. *Lines 106–116: there are results at the end of introduction?*
The methods or metrics such as "target overlapping coefficient" are not defined here! This paragraph is entirely impossible to read for a person who has not read the rest of the work before returning here. It should be removed!
Oh, one final thing on this part: the 9-degree error is NOT much lower than a 13-degree error. They are about equal, and both are HORRIBLY BAD VALUES for such a simple quantity that can be modelled with physics-based models instead of DNN.
13-degree error is comparable to a one-shell model, and 9-degree error to omitting the whole of white matter altogether (Stenroos & Koponen, 2019). This means that the "approximate DNN model with anisotropy" performs worse than much simpler and faster conventional physics-based models. This is despite requiring much more input data!

Considering this performance, or lack of thereof, the authors use FAR TOO BOLD words such as "the rich information" (line 114) and "ultra-short computation time" (line 114).
Further, if the computation time is from the extraordinarily expensive high-end GPU, it should not be used to describe the suitability to "clinical setting" (line 115).
PS. It is computation time, not computation time. This error repeats throughout the work.

We apologize about the confusions about the results in introduction. We removed the last paragraph of introduction about results.

Material and methods (at this point, I will shorten my observations, as I have written a tad overly long response already).
19. Line 124: define "the minimal processing pipeline" (reference or description)

We added more information and the reference about the preprocessing of diffusion MRI. The revised sentence is below:
The HCP diffusion MRI datasets were preprocessed with corrections for head motion and distortions and were co-registered with T1w MRI [28].

20. Line 125: define "the native space" (the native space of what, a description)

We meant to say the native space of the subject. For clarity, we changed it to "T1w MRI" as in our response to your previous comment.

21. Line 126: questionable use of SimNIBS. The VCM construction should be given both T1 and T2 images for better performance.

We agree that using T_{1w} and T_{2w} can enhance the accuracy of tissue segmentation, thus improving the accuracy of the simulation results. We will continue developing and training our DNN_S to make it possible to integrates both T_{1w} and T_{2w} MRI in our future work. We added more comments in Line 483 about the limitations of the method:
Second, the trained DNN models not only depend on the type of coils, imaging protocols but also the data processing methods. In particular, tissue segmentations in this study were obtained based on T1w MRI, but more accurate results can be obtained by using both T1w and T_{2w} MRI. Thus, further development and training of the DNN models are needed to integrate different tissue segmentation approaches for more accurate prediction results.

22. Line 126: Which SimNIBS version it was, and why there is no reference?

It was version 3.0.8. We added the information in Line 106 and Line 192.

23. Line 128: Unnecessary sentence "We note that another command headreco can also be used to generate head models which may lead to different simulation results [Nielsen et al. 2018]."

This sentence was removed.

24. Instead of unnecessary sentences like in point 23, the previous sentences should contain all relevant modelling parameters. (The work further glosses over the entire problem with selection of conductivity parameters, despite being about anisotropic conductivity, which is finicky to the choice of the isotropic baseline values.)

We added the following sentence in Line 113 about model parameters:
The scalar-valued tissue conductivity for white matter, gray matter, CSF, bone and scalp were set as 0.126, 0.275, 0.1654, 0.01 and 0.465 S/m, respectively, which were the default values in SimNIBS. Moreover, the distance from the coil to the scalp was 4 mm which was also the default value.

25. Line 137: You seem to provide exact details on version of MATLAB, but for some reason omit them from SimNIBS at all points.

The version number of SimNIBS 3.0.8 is added in Line 106 and Line 192. That was the latest version when we started our project and was used throughout the project.

26. Line 146: what does this sentence about the "average degree between the nearest handle directions being about 4.6 degrees" even mean?

We apologize for the confusion. The sentence in Line 119 was revised to below:
In order to generate E-field maps with different coil centers and orientations, we sampled the position of the coil from the positions of the EEG 10-10 system and with the coil handle directed to 78 different directions with approximately 4.6o angular resolutions.

27. Line 165: grossly incorrect statement. Just having the T1 image (or even the correct set for good VCM, i.e., T1 and T2) has nothing to do with not computing the direction of the E-field! Is this the reason for the ridiculous claim of point 15? The anisotropy has a (small compared to just the much larger isotropic conductivity differences) effect on the field directions. It is not a fundamental reason for the field directions. I refer now to sentence: "We note that the T1w MRI was used in [Yokota et al., 2019] to predict only the magnitude of the E-field since T1w MRI does not contain diffusion information about the axon orientation."

We meant to say that the diffusion tensors provide information about the direction of anisotropic tissue conductivity which is potentially helpful in the DNN method to enhance the precision of the predicted E-field. To avoid confusion, we removed this sentence.

28. Line 169: “field” not “filed”.

Fixed.

29. Line 169: I assume you refer to the spatial distribution of the change of the vector potential (dA/dt) at the beginning of the TMS pulse, and not peak temporal changes!

Corrected, see Line 163. Thanks.

30. Line 177-179: The authors critique of (Yokota et al., 2019) could also be said about their own work! Let me paraphrase: “Thus, the DNN in (THE AUTHORS) is not a solver for the forward model but also needs to learn the well-known underlying physics of electromagnetic induction, which may reduce the prediction accuracy.” This is basically my main critique of both this work and the work of (Yokota et al., 2019).
In their current state, both methods are FAR LESS accurate than any PHYSICS-BASED solvers. And, in the case of the present manuscript, they are also slower than properly implemented fast physics-based solvers. The approximation by (Yokota et al., 2019) was at least momentarily the fastest approach with more complex than spherical geometry, before being caught up and surpassed by the fast BEM solvers.

We have removed this sentence. We agree that the output of DNN is only an approximation of the simulation results. Here, we meant to point out a major difference between our method and the (Yokota et al., 2019) that our method used the dA/dt map as an input to the DNN. As for the prediction speed, the method in (Yokota et al., 2019) was devised to optimize prediction speed with smaller FOV and scalar output with the dimension of output data being 72x144x24x1, whereas our method used a much larger FOV with vector E-field prediction with the dimension of output data being 180x220x120x3 for whole-brain estimation and explored the difference between different types of images.

We added the following sentence in Line 141:
Different from the method in [21], the proposed DNNs not only predict vector E-field maps but also predict it in the whole brain as opposed to scalar E-field maps in a small region. In particular, the dimension of the output data of the proposed DNNs is 180x220x120x3, whereas dimension of the output of the method in [21] is 72x144x24x1.

31. But, then the positive. Line 194: you actually, should the result survive through the proper cross-validation against the error for a SimNIBS model without the DTI data, try to make things that are not possible with a physics-based model.
The ‘T1-20’ model approximates the DTI dataset without needing one.

This result, however, will need to be compared to the computation against a physics-based model without the DTI data. As, if the physics-based model has prediction errors smaller than 13 degrees, then it is both faster & more accurate than the proposed DNN-based model. Such comparison is missing, and without it, I will suggest rejection.
The approximation of the DTI data is only true if the 'T1-20' can perform better than just using a physics-based solver without the data. (For which the error is likely much less than 10 degrees.)

We appreciate your positive comment and insightful viewpoint. Yes, it is indeed a potential advantage of the DNN method to use T1w MRI to predict anisotropic conductivity-based results. Accordingly, we added the following sentence in Line 493:
It is also a potentially useful tool to use only anatomical images, e.g., T_{1w} MRI, to predict E-field based on anisotropic conductivity tensors when diffusion MRI is not available in clinical settings, though further improvements in prediction accuracy are needed.

Regarding the angular error, we previously computed the angular error in whole brain. Since the E-field in brain tissue far from the coil has much weaker magnitude, minor prediction error can lead to significant angular differences. Thus, we also added the evaluation for angular error in the target region, as shown in Table 2. The angular error for T1-aniso20 model is 6.605° in target regions which is similar to the 6.688° for the T1-iso20 model to predict E-field maps with isotropic conductivity. The angular error for the C-20 model is about 4.851°.

32. Line 231-233, rewrite this sentence to make sense of it.

The revised sentence is now in Line 186 as below:
We first initialized the network weights using the method proposed in [37]. We then used the RAdam (Rectified Adam) [38] optimizer for network training with modulate parameters β1 = 0.9, β2 = 0.999 and the initial learning rate lr = 0.002. Step learning rate strategy was employed with the initial lr decayed by gamma=0.5 after every 5 epochs.

33. Line 282: on scalp, not on skull.

Fixed. We changed "skull" to "scalp"

34. Line 293: between E-field distributions, not between the brain regions.

Fixed. We changed "brain regions" to "E-field distributions".

35. Line 303: why is the word reference in quotation marks? Remove them.

Fixed. The quotation marks were removed.

36. Line 306-310: This error metric is incorrect, and underestimates the relevant prediction error. The correct method is to compute the magnitude of the vector difference (similarly to previous works with physics-based models).

Your method gives an error of 0 even if you would have predicted the field direction incorrectly by 180 degrees. (See the point about testing the coil with a different type of TMS coil, such as circular coil.)

We separated evaluated the magnitude differences (MAE and MRE) and angular differences (MDE) between the predicted and simulated E-field distributions. Following your suggestion, we added a new measure, i.e., the normalized root mean-square error (NRMSE), to compare the vector E-field according to the following definition

$$NRMSE = \frac{1}{N}\sum_{1}^{N}\left(\frac{\|E_P - E_R\|}{\|E_R\|}\right),$$

where N denotes the number of voxels E_P and E_R denote the predicted and reference vector E-field. The equation was added in Line 293.

37. Line 324: Comparing apples to oranges. The computation speed for the DNN is told with two flagship GPUs, and the computation speed for the (not exactly optimized for speed) FEM is told with an undisclosed hardware (probably no GPU acceleration).

We removed the following sentence “on the other hand, the time needed to simulate the corresponding training data using FEM was about 30 to 60 second.”

38. Line 327: Repeating the incorrect claim that 240 ms computation is ‘real time”. The authors never mention what they actually mean with that. They give example of neuronavigation, for which such computation speed is too slow.

We removed the sentence: Thus, the trained neural network was able to significantly reduce the time for E-field simulation making it useful for real-time neuronavigation.

39. Line 339: Significantly as in statistical significance? Or, some other significance? If using the latter used criteria of visual indistinguishability, both are equally good, or rather bad. Further, line 342: I saw not even a statistically significant difference between the results of C-20 and C-60?

We revised these sentences to below:

Overall, the T1-iso20 and T1-aniso20 models had similar performances in predicting E-field maps with isotropic and anisotropic conductivity, respectively. The C-20 model overperformed the T1-iso20 model with higher TOC and correlation measures and lower EPD, MAE and MRE measures, indicating that conductivity maps provide better performance than T1w MRI to predict E-field maps. The C-60 model had similar

performance as the C-20 indicating that using datasets from 20 subjects can provide reliable training results which is consistent to conclusions in [21]. The performance of the C-60 model at non-EEG positions that were no included in the training dataset was similar to the performance at EEG positions. In particular, the EPD was about 1.3 mm indicating that C-60 provided high accuracy for localizing TMS targets on brain surfaces.

40. Table is on line 345: Why is the angle error omitted from this table?

Only the magnitude of E-field was mapped to brain surfaces. But the angular error was only evaluated in volumetric space as shown in Tables 2 and 3.

41. Line 354: what exactly do you mean with "almost visually indistinguishable"?

We meant to say that results in first column and the second column of Figure 3 were similar. We have removed this sentence to avoid confusions.

42. Line 362, figure 3: Is this the difference of fields or difference of magnitude of fields? If former, there is 10% errors in the fields? If latter, well…

The mean relative error was summarized in Table 1. Overall, the mean relative error is about 7.7% but some regions were about 10% or higher. We added the following sentence in Line 343:

Though the MRE was about 7.7% over the entire brain surface, the relative error in some regions was 10% or higher.

43. Line 382: The angle errors are humongous. They are comparable to far simpler head models, not to some simple approximation. Compared to the approximation errors due to the fast quadrature of (Stenroos and Koponen, 2020), where the angle error was < 0.1 degrees, the 9 degree and the 13 degree errors are unusable.

We added the following sentences in Line 463 regarding the limitations on angular error

The angular error of the C-20 and C-60 models in target regions were about 4.841 and 4.474 degrees which were lower compared to the 6.688 degree for T1-iso20 and 6.605 degrees for T1-aniso20. But the angular errors were much higher than the fast quadrature method [22]. Thus a different training strategy may be needed to improve the angular precision of the DNN models, which will be explored in our future work.

44. Line 393, table 2: the location error is also 10 mm even for the 'C-60'. Such larger error is unusable for neuronavigation! Also, based on this table, is the angle error rather not 14 degrees and not 13 degrees?

We notice that our previous approach for computing E-field peak distance in the volume space was sensitive to outliers. We improve the estimation algorithm by using the average value of 3x3x3 neighboring voxels to determine the position of peak values of the E-field

maps. For the non-focal E-field distributions corresponding to the Magstim-70mm-Circular coil, the target position was determined as the average coordinate of the top 200 voxels. Accordingly, we added the following sentence in Line 267:

To reduce the influence of outliers on EPD in the volume space, we took the average value of the E-field magnitude in 3x3x3 neighboring voxels within the gray matter region as magnitude of a voxel to determine the location of the peak value of E-field maps for Magstim-70mm-Fig8 and MagVenture-MC-B70 coils. For the less focal E-field corresponding to the Magstim-70mm-circular coil, the location of the target was determined as the average position of the top 200 voxels with the highest E-field magnitude in the gray-matter region.

The updated results are shown in Tables 1 to 3. We added the following sentences in Line 458 about the limitations for targeting accuracy:

The experimental results showed that the target overlap ratio of the C-60 model on brain surfaces was 95.6% and the E-field peak distance was 1.3 mm and the correlation coefficient was about 0.997. The distance error increased in the volume space to about 11.3 mm. The T1-iso20 and T1-aniso20 models had reduced targeting accuracy with the E-field peak distance on brain surfaces being 3.959 mm 3.736 mm, respectively, and 8.225 mm, 12.601 mm in volume spaces. The angular error of the C-20 and C-60 models in target regions were about 4.841 and 4.474 degrees which were lower compared to the 6.688 degree for T1-iso20 and 6.605 degrees for T1-aniso20. But the angular errors were much higher than the fast quadrature method [22]. Thus a different training strategy may be needed to improve the angular precision of the DNN models, which will be explored in our future work.

45. Line 407, table 3: the EPD, the correlation error, and the MDE are all unusably large! Such errors seem about the same as the error of a local sphere model? Compare to results of (Stenroos & Koponen, 2019).

The following sentence was added in Line 465:

But the angular errors were much higher than the fast quadrature method (Stenroos and Koponen, 2019). Thus, a different training strategy may be needed to improve the angular precision of the DNN models, which will be explored in our future work.

We note that the errors in (Stenroos & Koponen, 2019) were evaluated at much fewer positions, i.e., N=900, at cortical regions of interest. Thus, we think it should be a fair comparison with the surface-based evaluation in Table 1 where the correlation for C-60 was 0.997 and the distance error was 1.394 mm.

Discussion and conclusions:

46. Line 430: "novel advantages" what does this even mean?

We removed "novel" in the sentence.

47. Line 431: this is not a new feature: "First, it can be applied to predict E-field vector volumes with arbitrary coil positions and orientations"

Thanks. We revised the summary in Line 427 to below:

Our method has several major differences compared to the DNN method in [21]. First, our method uses the dA/dt map of the magnetic coil to predict vector E-field. Thus, the trained DNN model can be applied to predict E-field maps for different TMS coils. Second, we proposed a novel architecture of neural networks, i.e., 3D-MSResUnet, to improve the prediction accuracy by combination of residual module and multi-scale feature maps into 3D-U-net architecture. Third, our method used anisotropic conductivity maps to improve the prediction accuracy of E-field maps.

48. Line 433: with angle error close to 10 degrees, I would not exactly call this accurate: "to accurately predict the magnitude and the direction of E-field."

We removed "accurately" in the sentence.

49. And, then line 439, compare to point 31. Basically, you show that your DNN is worse than a physics-based model, and not fundamentally faster. "Our work shows that deep learning can be further used as a solver for the forward model for estimating whole-brain E-field in TMS."
I would like to point out that you have far more than 10 times more computational power than the fast-multigrid method by (Laakso and Hirata, 2012), which took just 3 s in (Yokota et al., 2019).

And, you do not even compare to such fast solver (~3 s), but rather compare your GPU-accelerated computation time to a not-optimized for real-time FEM without any GPU acceleration (30-60 s).

We removed this sentence. We agree that current prediction speed is far from being real-time. But it is not a fair comparison with the method by (Laakso and Hirata, 2012) and (Yokota et al., 2019) since our method predicts 3-dimensional E-field distributions within a much larger FOV. We believe the prediction speed of our method can be enhanced by using faster GPU computing resources or reducing the FOV or only predicting E-field on brain surfaces, which will be explored in our future work. We have added more comments in Line 473 about the prediction speed. The following are the added sentences:

The prediction of a whole-brain E-field volume using the trained neural networks took about 0.24 s. In practice, additional time is needed to apply rigid transformation to the dA/dt map according to the coil position which is expected to take much shorter computation time. Moreover, the prediction speed is still slower than the fast quadrature method [22] and the DNN based method [21] though the predicted E-field by these methods have relative lower dimensions. Further improvement in prediction speed in needed in situations when real-time visualization of E-field is required. For example, to predict 20 coil positions per second required the prediction time should be reduced to less than 0.05 s. A potential method to

improve the prediction speed is to reduce the field of view of the E-field or to only predict E-field on brain surfaces, which will be explored in our future work.

50. Line 444-448: No you did not show this. What was your reference model compared to which you showed that the FEM was accurate in its prediction? Did you test for the sensitivity of your solution to your input parameters (which you failed to disclose), or even just the model resolution?

We changed the discussion in Line 436 on conductivity map vs T1w MRI to below:
The experimental results show that the T1-aniso20 model can use T1w MRI to predict E-field maps based on anisotropic conductivity with similar performance as the T1-iso20 model for predicting E-field based on isotropic models. While using T1w MRI to predict E-field based on anisotropic models is an advantage compared to physics-based FEM method, the prediction accuracy is worse than the results of C-20 that uses anisotropic conductivity tensors for E-field prediction. But the dependence the C-20 and C-60 models on diffusion tensors limits their applications in situations when diffusion MRI is not available.

51. Line 467: 9 degree error in volume is not similar in direction. It is horribly badly off.
We removed the word "similar" and revised the sentence in Line 463 to:
The angular error of the C-20 and C-60 models in target regions were about 4.841 and 4.474 degrees which were lower compared to the 6.688 degree for T1-iso20 and 6.605 degrees for T1-aniso20. But the angular errors were much higher than the fast quadrature method [22]. Thus a different training strategy may be needed to improve the angular precision of the DNN models, which will be explored in our future work.

52. Line 470: 13.7 degrees is not "as low as" but rather horribly bad prediction.

We removed "as low as" and revised the sentences as provided in our response to 51.

53. Line 473: how is this work applicable to clinical setting? Where do we get all the DTI data, and the computational resources?

We added the following sentence in Line 438 regarding the limitations of C-20 and C-60 on the dependence of DTI and the potential advantage of using T1w MRI:
While using T1w MRI to predict E-field based on anisotropic models is an advantage compared to physics-based FEM method, the prediction accuracy is worse than the results of C-20 that uses anisotropic conductivity tensors for E-field prediction. On the other hand, the dependence the C-20 and C-60 models on diffusion tensors limits their applications in situations when diffusion MRI is not available.

54. Line 478: the computation time (not computation time) is not ultra-short.

We revised the paragraph on prediction speed. The updated text is below:

The prediction of a whole-brain E-field volume using the trained neural networks took about 0.24 s. In practice, additional time is needed to apply rigid transformation to the dA/dt map according to the coil position which is expected to take much shorter computation time. Moreover, the prediction speed is still slower than the fast quadrature method [22] and the DNN based method [21] though the predicted E-field by these methods have relative lower dimensions. Further improvement in prediction speed in needed in situations when real-time visualization of E-field is required. For example, to predict 20 coil positions per second required the prediction time should be reduced to less than 0.05 s. A potential method to improve the prediction speed is to reduce the field of view of the E-field or to only predict E-field on brain surfaces, which will be explored in our future work.

> *55. Line 480: visualization in real-time might indeed be useful (not quite essential), but your work fails to deliver such speeds, unlike previous 'real-time methods'*

The updated text was provided in our response to 54.

> *56. Line 483: Probably soon tenth repetition of this same line does not make it true. Please, do not oversell your work.*

The entire paragraph was revised with the updated sentences provided in our response to 54.

> *57. Line 487: Fundamental problem with understanding physics-based models. A FEM and a BEM will produce practically identical E-field predictions, e.g., (Gomez et al., 2020), and thus the DNN should not change based on which physics-based model it is trained on. This is the whole reason for physics-based models. We get the SAME result with a BEM as with a FEM, or an FDM (given adequate resolution for each).*
>
> *This is why DNN is also inherently problematic for this type of problem. You essentially try to make it learn very well-known physics, instead of actually just using said physics to compute the result.*

We meant to say that the DNN were trained to predict the E-field simulated with anisotropic conductivity. Since we have now added the results for isotropic conductivity, we revised the entire paragraph. Below is the updated text.

Finally, we note that the DNN methods have several limitations. First, the neural networks were trained to predict E-field simulated with fixed values for tissue conductivity, i.e., specific values of conductivity for different tissue types, a specific coil and specific imaging protocols, which are limitations compared to physics-based FEM algorithms. Second, the trained DNN models not only depend on the type of coils, imaging protocols but also the data processing methods. In particular, tissue segmentations in this study were obtained based on T1w MRI, but more accurate results can be obtained by using both T1w and T2w MRI. Thus, further development and training of the DNN models are needed to integrate

different tissue segmentation approaches for more accurate prediction results. Third, the DNN models were only trained based on data from health subjects whereas physics-based FEM algorithms have more broad applications for patients with tumors or lesions. Thus, the goal of the DNN-based method is not to replace FEM approaches. But the DNN-based methods can potentially be useful to accelerate the prediction of E-field in situations when their performances are validated.

58. Line 490: There were other far more important fixed parameters than distance to scalp (which your dA/dt –based approach should in any case be fairly robust on). But then again, this revisits points 31, 57, etc.

DNN is fundamentally wrong tool for solving the forward problem. (The 'T1-20' to predict the DTI-FEM result is interesting, however. That is, if you compare it to no-DTI FEM to see if it can actually perform better.)

The updated text is provided in the above response to 57. We appreciate your viewpoint on using T1w to predict DTI-FEM. Following your suggestion, we have trained a new model T1-iso20 to predict non-DTI FEM. We added the following sentence to point out a potential advantage of DNN:

It is also a potentially useful tool to use only anatomical images, e.g., T1w MRI, to predict E-field based on anisotropic conductivity tensors when diffusion MRI is not available in clinical settings, though further improvements in prediction accuracy are needed.

59. Line 490: for example, you now fix the conductivity parameters. And, of course you model only works for 'normal' brains. This makes the method unsuitable for, e.g., neuronavigation of persons with lesions etc. (which is where the physics-based models excel).

We agree. Below is the updated text in Line 488 regarding the limitations:

Third, the DNN models were only trained based on data from health subjects whereas physics-based FEM algorithms have more broad applications for patients with tumors or lesions

60. Line 490: based on what data is the DNN expected to be robust against the change of the long list of parameters you fail to mention at any point in the manuscript?

We removed this sentence.

Reviewer #2

This paper introduces a novel U-net architecture for realtime Efield analysis of TMS. The manuscript is well written and their methods technically sound. However, some important issues need to be addressed:

Since they are claiming this is accurate they should mathematically define all of their error metrics.

Thanks. We have defined each metrics. The target overlapping coefficient (TOC) was defined as

$$TOC = \frac{2TP}{2TP+FP+FN} \quad (1)$$

where the TP, FP and FN mean true positive, false positive and false negative between the prediction E-Field and the reference E-Field.

The E-field peak distance (EPD) was defined as

$$EPD = \|Peak(E_P) - Peak(E_R)\|, \quad (2)$$

where E_P and E_R denote the predicted and reference E-field and the $Peak()$ function obtains the coordinate of the voxel at the gray matter region or the vertex at brain surface with the maximum magnitude. To reduce the influence of outliers on EPD, we took the average value of the E-field magnitude in 3x3x3 neighboring voxels within the gray matter region as magnitude of a voxel to determine the location of the peak value.

The E-field similarity, i.e., correlation, was commutated as

$$Correlation = \frac{Cov(\|E_P\|, \|E_R\|)}{\sqrt{Var(\|E_P\|)Var(\|E_R\|)}}, \quad (3)$$

where $\|E_P\|$ and $\|E_R\|$ denote the magnitude of the predicted and the reference E-field, respectively.

The mean absolute error (MAE) and mean relative error (MRE) were defined as

$$MAE = \frac{1}{K}\sum_1^K \big|\|E_P\| - \|E_R\|\big|, \quad (4)$$

$$MRE = \frac{1}{K}\sum_1^K \left(\frac{\big|\|E_P\| - \|E_R\|\big|}{\|E_R\|}\right), \quad (5)$$

where K denotes the number of vertices on the surface.

Following your comment 1) below and Comment # 36 of Reviewer #1, we added the following normalized root-mean-square error (NRMSE) to compare vector E-field maps:

$$NRMSE = \frac{1}{N}\sum_1^N \left(\frac{\|E_P - E_R\|}{\|E_R\|}\right), \quad (6)$$

where N denotes the number of voxels.

The mean directional error (MDE) was defined as

$$MDE = \frac{1}{N}\sum_1^N acos\left(\frac{E_P \cdot E_R}{\|E_P\|\|E_R\|}\right). \quad (7)$$

1) *The authors do not compare to standard error metrics like L2, pointwise error like in https://doi.org/10.1016/j.brs.2019.09.015 . Also, others like RDM. What is the relative performance to existing methods.*

Thanks for pointing out this metric. We added the normalized root mean-squared error (NRMSE) metric provided above which is the same as the L2 metric in the suggested reference.

We computed NRMSE for whole-brain and the target regions. The updated results are provided in Tables 2 and 3.

2) *How long does it take to compute dA/dt?;this should be included in computation time.*

Thanks for your comment. dA/dt is provided from the selected coil. A rigid transformation is needed to transform the dA/dt to the selected position. Since the transformation parameters are provided based on the coil position, the rigid transformation takes much shorter computation time than the prediction time. We added the following sentence in Line 470:
In practice, additional time is needed to apply rigid transformation to the dA/dt map according to the coil position which is expected to take much shorter computation time.

3) *This method has not been trained with different coils, how do we know the trained network will generalize to other coils?*

The DNNs were trained based on Magstim-70mm figure-8 coil. We tested its performance for MagVenture MC B70 (figure-8) coil and Magstim circular 70 mm coil. The results are provided in Table 3, see below.

We note that the DNNs can be further trained using the simulated data from different coils. Results from this work can be used as initial parameters to expedite the training procedure.

4) *In a sense when you move the coil the dA/dt is moved; this is equivalent to moving the brain. So their claim that passing these maps changes something needs a deeper rationale.*

A main difference between our method and (Yokota et al. 2019) is that our method uses dA/dt map as an additional input to predict the E-field whereas (Yokota et al. 2019) only uses the coil position as input. Since the two coils can be placed at the same position to generate different E-field, the method by (Yokota et al. 2019) intrinsically require different DNN models for different coils. An advantage of using dA/dt map to predict E-field is that the dA/dt maps can characterize differences between the coils. Thus, a single DNN model can be potentially applied to multiple coils. We added the first sentences in Line 215:
We note that a major difference between the proposed approach and the method in [21] is the inclusion of the dA/dt map as a input to the DNN. Thus, the DNN model can produce different E-field maps for different coils at the same positions. In our experiment, the DNNs

were trained based on simulated E-field using a Magstim-70mm-Figure8 [39] TMS coil. To examine the performance for other coils, we applied the C-60 model to predict E-field maps for the MagVenture-MC-B70 coil, which has a similar Figure-of-Eight shape as the trained coil and the Magstim-70mm-Circular coil [40].

5) *is the DNN computing both primary plus secondary E-field or just secondary?*

It is the total E-field simulated using the finite-element method provided by the SimNIBS software.
