# Supplementary material for: Rapid whole-brain electric field mapping in transcranial magnetic stimulation using deep learning
Source: PLoS One. 2021 Jul 30;16(7):e0254588. doi: 10.1371/journal.pone.0254588 (PMC8323956; doi:10.1371/journal.pone.0254588)
Supplement: Attachment — Submitted filename: Response to Reviewers.pdf [file pone.0254588.s004.pdf]

Dear Editor,

We thank you for the opportunity to submit a revision of our manuscript (#PONE-D-20-30151). We are grateful for the reviewers' insightful and detailed comments which were helpful to improve the quality of this paper. Following your suggestions, we have added the suggested references by Reviewer #4 as reference [45], [46] and added more discussions about the future directions to improve the DNN based method. We have individually addressed the reviewers' comments and concerns.

Our responses are given in blue and the revisions in the manuscript are highlighted in yellow. We hope that these changes meet and exceed your expectations for publication.

On behalf of myself and all the co-authors.

Sincerely,

Lipeng Ning, PhD.
Assistant Professor
Department of Psychiatry
Brigham and Women's Hospital
Harvard Medical School

Reviewer #3:

In this study, authors propose a method to estimate the induced E-field from the magnetic field generated by TMS coil and the MRI image, using the framework of deep learning. Since the E-field can be estimated by the forward calculation of DNN without solving the problem, the estimation can be significantly accelerated than the FEM. In the experiment, the proposed DNN is evaluated by several metrics, and the effectiveness of the proposed method is confirmed.

To my understanding, the most relevant paper to this study is [21] by Yokota et al at 2019. In terms of TMS-induced E-field estimation using DNN, this study can be considered as a kind of next step of original one [21]. The main new contributions are three folds: (1) estimation of a whole brain region, (2) vector field estimation, and (3) magnetic filed representation of TMS coil parameters.

Contributions (1) and (2) are relatively weak because both vector and whole brain extensions are straightforward by introducing some powerful computational resources. On the contrary, since weak E-fields is often majority in whole brain, the necessity of whole region estimation is unclear for TMS targeting. Moreover, the requirement of the large DNN model with powerful computation (i.e., large memory of GPU) reduces practical usefulness. That is always trade-off.

The contribution (3) is important and it make the paper acceptable in my opinion. By introducing magnetic field representation of TMS coil parameter, the DNN can be trained and applied for various types of coils. It is nice that some computational experiments have conducted by using three different coils. However, it is necessary to care that the overfitting is more easily cased in the proposed framework because input space is quite larger than original one [21].

We appreciate your approval to our work. Your comments and suggestions have been tremendously helpful for us to improve the quality of our paper.

Reviewer #4:

Comments on the paper "Rapid whole-brain electric field mapping in transcranial magnetic stimulation using deep learning"

This is a modeling study which aims to use a neural network (DNN) to predict the TMS fields in subjects in a real time. It essentially develops the idea of Ref. [21] with an extra inclusion of the primary TMS field as an input. 65 Connectome subjects and the SimNIBS FEM solver have been used to perform numerical experiments.

While the original idea is interesting, the reviewer still tends to agree with the previous review #1 saying that "The DNN is inherently problematic for this type of problem".

The first reason is that it is obviously hardly possible to replicate a unique gyral pattern of a subject which, in its turn, uniquely defines the resulting TMS field, based on limited numbers of computations for other subjects (20 or 60 in the paper). Even for those numbers, training the DNN takes weeks, according to the paper, and requires a specially equipped workstation.

Thanks for your comments. Reviewer #3 also had a similar question in the previous review cycle that if DNN can be used to predict E-field using input data that is not like the training data. Our results in the third column of Table 3 have shown that the DNN trained based on a Fig-8 coil can be used to predict E-field for circular coils with reasonable performance. Moreover, the results in Table 2 have also shown that using 20 subjects can achieve similar performance as using 60 subjects. Thus, a suitably trained DNN can be reliable applied to data that not included in the training set. We agree that the long training time is a limitation of this method. But the once the DNN is trained, it can be applied to standard workstations to accelerate the E-field prediction. In our future work, we will develop more effective network structure to accelerate the training and prediction time of DNN.

The second reason is that, according to the manuscript, after the several weeks (!) of training, the approximate TMS field could be predicted in 0.24 sec. However, the paper does not compare this finding to the performance of the modern physical solvers, which estimate the TMS fields precisely.

As a first example, a new precise physical approach of Gomez et al [Gomez LJ, Dannhauer M, Peterchev AV. Fast computational optimization of TMS coil placement for individualized electric field targeting. NeuroImage. 2021 Mar;228:117696. doi: 10.1016/j.neuroimage.2020.117696.] allows us to predict the E-fields generated in an MRI-derived head model when the coil is placed at 5900 different scalp positions and 360 coil orientations per position (over 2.1 million unique configurations) under 15 min on a standard laptop computer. This performance of the exact FEM physical solver simply cannot be matched to the approximate results reported by the authors.

As a second example, another new physical approach of Daneshzand et al [Daneshzand M, Makarov SN, de Lara LIN, Guerin B, McNab J, Rosen BR, Hämäläinen MS, Raij T, Nummenmaa A. Rapid computation of TMS-induced E-fields using a dipole-based magnetic stimulation profile approach. Neuroimage. 2021 Apr 30:118097. doi: 10.1016/j.neuroimage.2021.118097.] allows computation of the E-field in ~100 ms given ~5 hours of preprocessing time. This performance of the exact BEM physical solver cannot again be matched to the approximate results reported by the authors.

Compared to these studies, the suggested approach seems to be a step back as being less accurate and significantly more lengthy.

Thanks for your comments and suggested references. In the work of [Gomez et al, NeuroImage, 2021], the fast computational algorithm estimates the E-field in a selected ROI to optimize the coil optimization. In [Daneshzand et al., NeuroImage, 2021], the E-field map is predicted on a surface mesh in ~100 ms but with about 5 hours preparation time. Compared to these methods, the merits of our DNN-based method lie in the simplification in data preprocessing since it does not need mesh models and the acceleration in whole-brain E-field volume prediction. But significant improvement is needed to improve the architecture and the training scheme of the DNN to improve its prediction speed and flexibility. In our future work, we will future develop the DNN method to predict E-field in a selected ROI or brain surface mesh. We expect that reduced data dimension and simplified network architecture can significantly reduce the prediction time. We added the following sentences in Line 475 to 487.

More recently, a fast computational algorithm was introduced in [45] to estimate E-field in a selected ROI so that the E-fields generated by coils placed at 5900 different scalp positions and 360 orientation per position can be computed under 15 minutes. In [46], a

rapid algorithm was introduced to compute E-field in ~100 ms on a cortical surface mesh with 120k facets and with about 5 hours of preparation time. Compared to these methods, the merits of our DNN-based method lie in the simplification in data preprocessing since it does not need mesh models and the acceleration in whole-brain E-field volume prediction. But significant improvements are needed to accelerate the prediction of E-field in target ROI to optimize coil positions and to achieve real-time prediction on brain surfaces. For this purpose, we will improve the architecture and training approach of the DNN in our future work to directly predict E-field in a selected ROI or on brain surfaces. We expect that reduced data dimension and simplified network architecture can significantly reduce the prediction time.

Minor comment:

1. Why is Ernie (one model) compared to the entire HCP dataset?

The Ernie model is used as an example to illustrate the feasibility and performance of DNN on a typical clinical dataset with different resolutions.
